# Body Composition and Pulmonary Function in Cystic Fibrosis

**DOI:** 10.3389/fped.2014.00033

**Published:** 2014-04-15

**Authors:** Saba Sheikh, Babette S. Zemel, Virginia A. Stallings, Ronald C. Rubenstein, Andrea Kelly

**Affiliations:** ^1^Division of Pulmonary Medicine, Cystic Fibrosis Center, Perelman School of Medicine at the University of Pennsylvania, Philadelphia, PA, USA; ^2^Department of Pediatrics, Perelman School of Medicine at the University of Pennsylvania, Philadelphia, PA, USA; ^3^Division of Gastroenterology, Hepatology and Nutrition, Perelman School of Medicine at the University of Pennsylvania, Philadelphia, PA, USA; ^4^Division of Endocrinology and Diabetes, The Children’s Hospital of Philadelphia, Philadelphia, PA, USA

**Keywords:** body mass index, lean body mass, fat mass index, lean body mass index, pancreatic insufficient

## Abstract

**Background:** Lower body mass index (BMI) is associated with worse pulmonary function in cystic fibrosis (CF). Hypothesis: lean body mass (LBM) is more strongly associated with pulmonary function than BMI is.

**Methods:** Anthropometrics, body composition by dual x-ray absorptiometry, and pulmonary function were determined in pancreatic insufficient CF (PI-CF) youth. Sex and age-adjusted *Z*-scores (BMI-*Z*, LBMI-*Z*, FMI-*Z*) were generated for CF and controls. (1) Associations of BMI-*Z* with LBMI-*Z* and FMI-*Z* and (2) age-adjusted associations of BMI-*Z*, LBMI-*Z*, and FMI-*Z* with FEV_1_%-predicted were tested.

**Results:** Two hundred eight PI-CF subjects had lower BMI-*Z*, LBMI-*Z*, and FMI-*Z* compared to 390 controls. BMI-*Z* was associated with lower LBMI-*Z* (*p* < 0.0001) in PI-CF. In females, LBMI-*Z* and BMI-*Z* were positively associated with FEV_1_%-predicted; this relationship did not persist for FMI-*Z* after adjustment for LBMI-*Z*. In males, only LBMI-*Z* and BMI-*Z* were associated with FEV_1_%-predicted.

**Conclusion:** In PI-CF youth, deficits in LBM were apparent. At lower BMI percentiles, BMI may not accurately depict LBM in PI-CF. In under-nourished PI-CF youth, this preservation of FM in preference to LBM is relevant since LBMI-*Z*, but not FMI-*Z*, is positively associated with FEV_1_%-predicted. Lean body mass index is more strongly associated with lung function compared to BMI, especially in the under-nourished child and adolescent with PI-CF.

## Introduction

Stature, weight, and body mass index (BMI) are typically used to assess nutritional status in cystic fibrosis (CF) since these measures are obtained in clinical practice and reference data are available. Moreover, since poor nutritional status is associated with worse pulmonary function in CF (1–3), the CF foundation (CFF) clinical care guidelines set a goal to maintain BMI above 50th percentile in children and above 22 or 23 in adults with CF in order to support optimal pulmonary function (4).

The association of higher BMI with better pulmonary function in CF is hypothesized to reflect the impact of lean body mass (LBM) upon respiratory muscle strength and physical well-being (5, 6). However, in the general population, BMI is used to indicate body fatness. Studies directly measuring body composition in CF are limited (5, 7, 8). Their results are difficult to generalize in children and adolescents with CF due to inclusion of adults, differing body composition methodologies, small sample sizes, and variability in the degree of malnutrition and pulmonary disease in the subject cohorts. Lower fat free mass (FFM), a measure of both bone and muscle mass derived from dual x-ray absorptiometry (DXA), indirectly estimates LBM and has been associated with lower FEV_1_%-predicted and more frequent pulmonary exacerbations in adults with CF (9–11). Adults with unrecognized FFM depletion, defined as normal BMI and low FFM, have lower FEV_1_%-predicted than adults with both normal BMI and normal FFM (12). This finding highlights the limitation of BMI as an informative marker of nutritional status in complex chronic diseases such as CF (5). Similarly, DXA-derived LBM, which estimates non-bone LBM, was correlated with degree of lung function impairment in adults and with clinical severity score in both children and adults in studies with small sample sizes (7, 13). In contrast, Williams et al. (14) studied body composition in 6- to 12-year-olds with PI-CF using a four-compartment model, a technique limited to the research setting. Their study found that girls with PI-CF had a significantly lower fat mass (FM) compared to age-, height-, and puberty-matched controls, and that FM was positively associated with FEV_1_%-predicted. This association was not significant in males and no association was seen with FFM in this cohort.

The extent to which worse nutritional status directly contributes to worse pulmonary function has not been fully delineated. Given the emphasis placed upon optimizing nutritional status in CF clinical care, interventions aimed at improving BMI, and an emergence of obesity (15) in children and adults with CF, an improved understanding of body composition especially fat compared to muscle stores in CF health is indicated. The aim of this study was to assess the associations of BMI and DXA-derived measures of LBM and FM with lung function in a population of children and adolescents with pancreatic insufficient CF (PI-CF) without significant CF-related morbidities. We hypothesized that the association of BMI and pulmonary function reflects the association of LBM and pulmonary function.

## Materials and Methods

### Subjects

Individuals with PI-CF, aged 5–21 years were recruited from the Cystic Fibrosis Centers at The Children’s Hospital of Philadelphia, Philadelphia, PA, USA, the Hospital of the University of Pennsylvania, Philadelphia, PA, USA, and multiple other CF Centers in the United States (refer to acknowledgments). Data were collected during the period from November 2000 to February 2002 for a study on bone mineral content (16) and then again at baseline in a separate cohort during a clinical trial from March 2007 to May 2011. The diagnoses of CF and pancreatic insufficiency were based upon CFF recommendations.

Inclusion criteria included an FEV_1_%-predicted >40%. Subjects were excluded for diabetes, cirrhosis or portal hypertension, history of lung or liver transplant, or presence of other medical conditions not associated with CF that could potentially affect growth.

A group of 390 healthy Caucasian children and young adults, aged 5–21 years were selected from the Philadelphia area as a control group (16). A separate group of 462 non-African American subjects were evaluated at our center to generate contemporary reference data for growth and body composition (17); standard deviation scores for CF subjects and healthy controls were generated from these reference data.

For CF subjects, medical records were reviewed and data were collected for genotype. CF genotype was categorized as homozygous for ΔF508 mutation, compound heterozygous with one ΔF508 allele, or other variants.

### Anthropometry

Weight was measured using a digital scale (Scaltronix, White Plains, NY, USA). Height was measured using a stadiometer (Holtain, Crymych, UK). BMI (weight/height^2^) was calculated. Age- and sex-adjusted *Z*-scores for height, weight, and BMI were calculated using current reference data (18).

### Puberty staging

Puberty status was ascertained using a validated self-assessment questionnaire to categorize Tanner stages (TS) of pubic hair distribution (both sexes), and either genital development (males) or breast development (females) (19).

### Dual energy x-ray absorptiometry

Spine and whole body DXA scans were acquired using a fan beam array (Hologic Delphi, Bedford, MA, USA), and analyzed using the Discovery software (version 12.3). Whole body scans were analyzed to generate estimates of LBM (kilogram) and FM (kilogram).

Lean body mass index (LBMI = LBM/height^2^) and fat mass index (FMI = FM/height^2^) were calculated for the CF and controls. Age-based reference curves were generated for males and females from 462 healthy subjects using the lambda-mu-sigma (LMS) method (20). LBMI and FMI were then converted to sex- and age-adjusted *Z*-scores for the CF and controls using these contemporary reference data.

### Spirometry

To assess pulmonary function, standard spirometry was performed by subjects with CF. FEV_1_ was reported as the percentage of predicted value (FEV_1_%-predicted) based upon prediction equations of Wang and Hankinson (21, 22). Forced vital capacity (FVC) was similarly reported as percentage of predicted value (FVC%-predicted).

### Statistical analyses

Continuous variables were summarized using mean, median, and standard deviation, and categorical variables were described using proportions. Means for normally distributed data including age, FEV_1_%-predicted, FVC%-predicted, BMI-*Z*, LBMI-*Z*, and FMI-*Z* were compared using two-sided *t*-test; *p* < 0.05 was considered statistically significant.

Linear regression was used to compare the relationships between BMI-*Z* and (1) LBMI-*Z* and (2) FMI-*Z* in CF and controls; since the relationship between BMI-*Z* and FMI-*Z* is non-linear, BMI-*Z* squared was included in the FMI-*Z* model. The interaction between BMI-*Z* and CF status was also assessed.

The analyses were stratified by sex and multiple linear regression was used to assess the associations between body composition (BMI-*Z*, LBMI-*Z*, and FMI-*Z*) and pulmonary function (FEV_1_%-predicted and FVC%-predicted). These associations were tested individually and in combined models. The fit of each model was evaluated by comparing the adjusted *R*^2^, likelihood ratio, and Akaike information criterion (AIC). Models were adjusted for age and age squared to account for the known age-related decline in pulmonary function. The effects of puberty [pre-pubertal (Tanner 1) vs. pubertal (Tanner 2–5)], race/ethnicity, type of CFTR mutation, and colonizing pathogens in sputum culture on these associations were tested. To test for effect modification by nutritional status, subjects were also categorized by BMI, acceptable (BMI ≥ 50th‰) vs. sub-optimal BMI ( <50th‰) (4).

The ability of BMI-*Z* < 0 vs. LBMI-*Z* < 0 to identify subjects with FEV_1_%-predicted ≥80 and <80% adjusted for age and sex was compared using logistic regression.

Regression diagnostics were performed on all models through graphical checks, the Shapiro–Wilk test of normality of the residuals, and the Cook–Weisberg test for heteroscedasticity. All analyses were performed using STATA 12 (StataCorp LP, College Station, TX, USA).

The Institutional Review Boards at each of the participating institutions approved the study protocols, under which these data were collected. Informed consent or assent was obtained as appropriate.

## Results

### Population characteristics

Characteristics for subjects with CF and PI are summarized in Table 1. A total of 211 subjects (114 males, 97 females), aged 5–21 years with PI-CF were evaluated. Spirometry was not available for three subjects. Data from the remaining 208 subjects were analyzed. Consistent with the known genetic epidemiology of CF, the majority (90%) were Caucasian, and 72% had at least one ΔF508 CFTR mutation.

**Table 1:** **Characteristics of subjects (mean ± SD)**.

	Males			Females		
	CF (*n* = 111)	Controls (*n* = 182)	*p*-Value	CF (*n* = 97)	Controls (*n* = 208)	*p*-Value
Age	12.3 ± 3.6	11.7 ± 3.6	0.22	12.5 ± 4.1	12.1 ± 3.5	0.35
HT-*Z*	−0.46 ± 0.91	0.18 ± 0.86	<0.0001	−0.46 ± 0.99	0.19 ± 0.82	<0.0001
WT-*Z*	−0.51 ± 0.90	0.24 ± 0.89	<0.0001	−0.44 ± 0.95	0.27 ± 0.80	<0.0001
BMI-*Z*	−0.33 ± 0.83	0.16 ± 0.96	<0.0001	−0.20 ± 0.88	0.23 ± 0.84	0.0001
LBMI-*Z*	−0.63 ± 0.94	0.01 ± 1.0	<0.0001	−0.56 ± 1.00	0.01 ± 0.97	<0.0001
FMI-*Z*	−0.37 ± 0.87	0.00 ± 1.0	0.0016	−0.39 ± 1.00	−0.02 ± 0.99	0.003
FEV_1_%	90 ± 20	NA^a^	–	88 ± 21	NA	–
FVC%	96 ± 18	NA	–	91 ± 21	NA	–
Race
Caucasian	101 (91%)	182 (100%)		85 (88%)	208 (100%)	
Other	9 (8%)	0		8 (8%)	0	
Unknown	1 (1%)	0		4 (4%)	0	
Pubertal/pre-pubertal	75/36	113/69	0.88	73/24	154/54	0.47

Males and females with CF were similar in age, and the various pubertal stages were well-represented in both sexes. Mean growth and body composition *Z*-scores were all <0 in the CF population. No sex differences in weight-*Z*, height-*Z*, BMI-*Z*, LBMI-*Z*, and FMI-*Z* overall, or within puberty groups (pre-pubertal or pubertal) were identified (data not shown).

**Figure 1:**
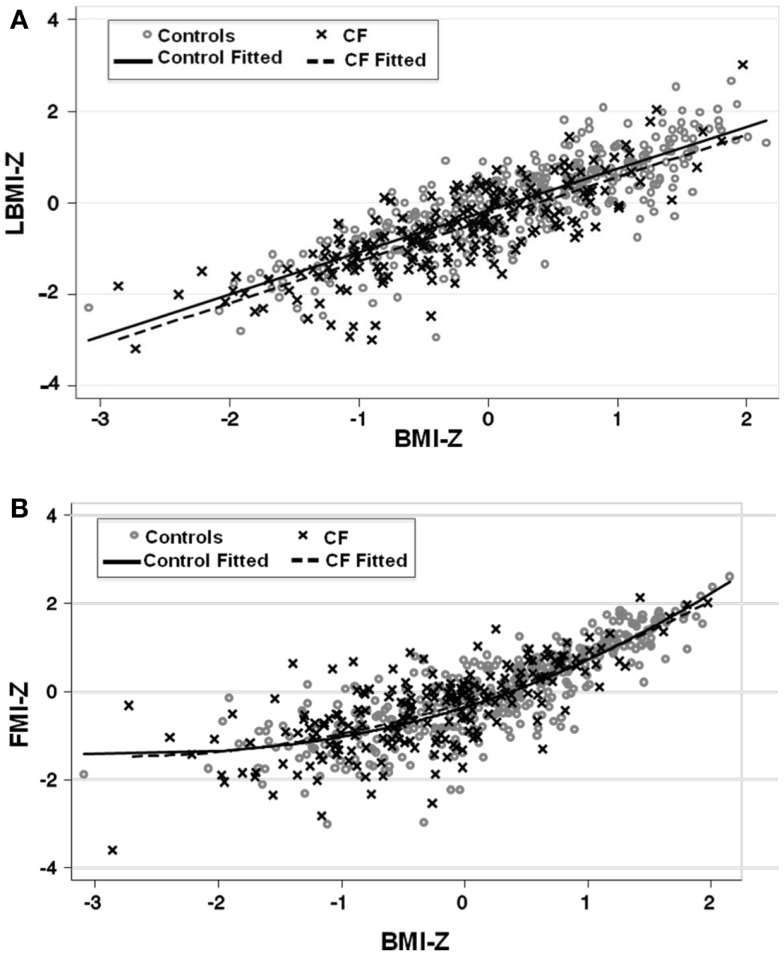
**(A)** The association between LBMI-*Z* and BMI-*Z* among healthy controls and patients with CF. There is a positive association between LBMI-*Z* and BMI-*Z* in all patients. Patients with CF have lower LBMI-*Z* for BMI-*Z* compared to controls. **(B)** The association between FMI-*Z* and BMI-*Z* among healthy controls and patients with CF. There is a positive association between BMI-*Z* and FMI-*Z* in all patients that is not altered in the presence of CF. Circles represent data points for controls and the solid line represents the fitted line for controls. Crosses represent data points for CF patients and the dashed line represents the fitted line for CF subjects.

Lean body mass index-*Z* was positively associated with height-*Z* (*p* < 0.0001) but the relationship was attenuated in the setting of CF (*p* < 0.0001). LBMI-*Z* was also positively associated with weight-*Z* (*p* < 0.0001) with a minor interaction between weight-*Z* and CF status (*p* = 0.06) such that for a given weight-*Z*, CF subjects have a lower LBMI-*Z* compared to controls. Similarly, LBMI-*Z* was positively associated with BMI-*Z* in both CF and controls, but this positive relationship was attenuated in the setting of CF (*p* < 0.0001, Figure 1A and Table 2), even after adjustment for puberty. Moreover, CF subjects with “sub-optimal” BMI (*n* = 131) had lower mean LBMI-*Z* compared to controls with BMI < 50% (*n* = 154) (*p* = 0.006).

**Table 2:** **Association between BMI-*Z* and (1) LBMI-*Z* and (2) FMI-*Z* in CF subjects (*n* = 208) and controls (*n* = 390) using linear regression**.

Predictor variable	Partial β coefficient	95% CI	*p*-Value	*R*^2^
**OUTCOME VARIABLE: LEAN BODY MASS INDEX-*Z***				
Weight-*Z*	0.86	0.79, 0.95	<0.0001	0.57
Presence of CF	−0.04	−0.16, 0.09	0.5	
Weight-*Z* × presence of CF	−0.12	−0.25, 0.01	0.06	
BMI-*Z*	0.94	0.89, 0.99	<0.0001	0.69
BMI-*Z*	0.92	0.86, 0.97	<0.0001	0.70
Presence of CF	−0.18	−0.28, −0.08	<0.0001	
BMI-*Z*	0.92	0.85, 0.98	<0.0001	0.70
Presence of CF	−0.18	−0.28, −0.08	<0.0001	
BMI-*Z* × presence of CF	−0.00004	−0.11, 0.11	0.99	
**OUTCOME VARIABLE: FAT MASS INDEX-*Z***				
Weight-*Z*	0.76	0.70, 0.83	<0.0001	0.47
Weight-*Z* squared	0.14	0.10, 0.18	<0.0001	
Presence of CF	0.15	0.02, 0.28	0.024	
BMI-*Z*	0.88	0.83, 0.94	<0.0001	0.65
BMI-*Z* squared	0.17	0.13, 0.21	<0.0001	
BMI-*Z*	0.89	0.84, 0.94	<0.0001	0.65
BMI-*Z* squared	0.17	0.13, 0.22	<0.0001	
Presence of CF	0.06	−0.04, 0.16	0.24	

The positive association between FMI-*Z* and height-*Z* was blunted in the presence of CF (*p* = 0.001). In contrast, the positive relationship of FMI-*Z* with weight-*Z* and weight-*Z* squared was magnified in the presence of CF (*p* = 0.024). FMI-*Z* was positively associated with BMI-*Z* (*p* < 0.0001) in CF and controls (Table 2) after adjustment for puberty, but the relationship was not altered by the presence of CF (Figure 1B).

### Pulmonary function and body composition

Cystic fibrosis subjects had a mean FEV_1_%-predicted 89 ± 21, and a mean FVC%-predicted 96 ± 19. No difference in pulmonary function was found between males and females with CF (*p* = 0.41). Increasing age was associated with lower FEV_1_%-predicted (β-coefficient = −2.1, CI: −2.8, −1.4, *p* < 0.0001, *R*^2^ = 0.15). This negative association was more pronounced in females than males (decrease in FEV1% of 2.7 vs. 1.4% for each 1 year increase in age).

#### Females

**Figure 2:**
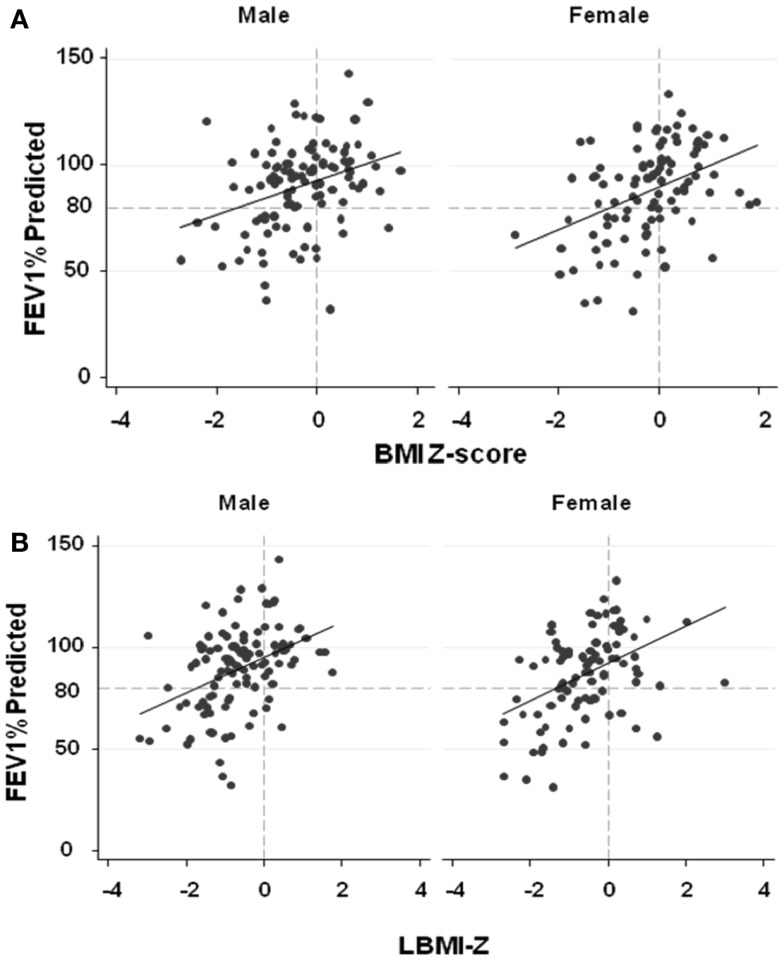
**(A)** The associations between FEV1%-predicted and BMI-*Z* adjusted for age among males and females with CF. BMI-*Z* is positively associated with FEV1%-predicted in males (*p* < 0.0001) and females (*p* = 0.001) with CF. **(B)** The association between FEV1%-predicted and LBMI-*Z* adjusted for age among males and females with CF. LBMI-*Z* is positively associated with FEV1%-predicted in males (*p* < 0.0001) and females (*p* < 0.0001). **(C)** The association between FEV1%-predicted and FMI-*Z* adjusted for age among males and females with CF. No association between FEV1%-predicted and FMI-*Z* was found in males (*p* = 0.26) while FMI-*Z* was positively associated with FEV1%-predicted in females (*p* = 0.046) with CF.

After adjusting for age, BMI-*Z* (*p* = 0.001), LBMI-*Z* (*p* < 0.0001), and FMI-*Z* (0.046) were all positively associated with FEV_1_%-predicted in females (Figure 2; Table 3). The relationship was strongest with LBMI-*Z* (lowest AIC), and the association of FMI-*Z* with FEV_1_%-predicted was no longer significant when included in the model with LBMI-*Z* (*p* = 0.96). Pubertal status did not alter the relationship of body composition and FEV_1_%-predicted (*p* = 0.10). The relationship between LBMI-*Z* and FEV_1_%-predicted was modified by nutritional status: in the setting of “acceptable” BMI-*Z* ( ≥0) the effect of increasing LBMI-*Z* on FEV_1_%-predicted was blunted (partial β-coefficient = −11.9; *p* = 0.008).

**Table 3:** **Relationship of body composition and pulmonary function in CF**.

Model	Predictor variable	Partial β coefficient	*p*-Value	*R*^2^		AIC
FEV_1_% PREDICTED						
Females	Age	−2.3	<0.0001	0.34		834
	BMI-*Z*	7.5	0.001	
	Age	−2.4	<0.0001	0.38		811
	LBMI-*Z*	8.0	<0.0001	
	Age	−2.2	<0.0001	0.44	LR χ^2^ = 7.49	806
	LBMI-*Z*	11.7	<0.0001		*p* = 0.006	
	Optimal BMI^a^ × LBMI-*Z*	−11.9	0.008			
	Age	−2.4	<0.0001	0.26		827
	FMI-*Z*	4	0.046		
	Age	−2.4	<0.0001	0.37		814
	LBMI-*Z*	7.9	<0.0001	
	FMI-*Z*	0.1	0.96	
Males	Age	9.46	0.004	0.23		960
	Age^2^	−0.42	0.001	
	BMI-*Z*	7.8	<0.0001	
	Age	11.78	<0.0001	0.32		928
	Age^2^	−0.51	<0.0001	
	LBMI-*Z*	9.77	<0.0001	
	Age	12.03	<0.0001	0.31	LR χ^2^ = 0.18	932
	Age^2^	−0.52	<0.0001		*p* = 0.67	
	Optimal BMI^a^ × LBMI-*Z*	−2.2	0.68			
	Age	8.85	0.013	0.12		967
	Age^2^	−0.39	0.004	
	FMI-*Z*	−2.32	0.28	
	Age	11.67	<0.0001	0.33		929
	Age^2^	−0.50	<0.0001	
	LBMI-*Z*	9.80	<0.0001	
	FMI-*Z*	−2.48	0.18	

*^a^BMI less than (or) greater than or equal to 50 percentile*.

Moreover, after adjustment for age, females with “acceptable” LBMI-*Z* (*Z* ≥ 0 or LBMI ≥ 50th‰) were five times more likely to have an FEV_1_%-predicted >80% than females with low LBMI-*Z* ( <0) (OR = 5.1, *p* = 0.016) while females with acceptable BMI-*Z* ( ≥0 or BMI ≥ 50th‰) were about six times more likely to have an FEV_1_%-predicted >80% (OR = 6.3, *p* = 0.002) (Table 3).

Body mass index-*Z* was positively associated with age-adjusted FVC%-predicted in females (partial β-coefficient = 4.6, *p* = 0.046). Similarly, after adjustment for age, LBMI-*Z* was positively associated with FVC%-predicted (partial β-coefficient = 5.5, *p* = 0.004). In contrast, FMI-*Z* was not associated with FVC%-predicted (*p* = 0.11).

#### Males

As shown in Figure 2 and Table 3, BMI-*Z* (*p* < 0.0001) and LBMI-*Z* (*p* < 0.0001), but not FMI-*Z*, were positively associated with FEV_1_%-predicted after adjustment for age in males. Based on AIC, LBMI-*Z* was a better model than BMI-*Z*. Inclusion of FMI-*Z* in the model with LBMI-*Z* did not improve the model, and the association of FMI-*Z* with FEV_1_%-predicted remained insignificant (*p* = 0.18). No interaction was found between LBMI-*Z* and BMI% category ( <50 vs. ≥50‰) in males (*p* = 0.68). Pubertal status did not alter the relationship of body composition with FEV_1_%-predicted in this male CF population (*p* = 0.41).

After age adjustment, males with acceptable LBMI-*Z* ( ≥0) were about five times more likely to have an FEV_1_%-predicted >80% than males with low LBMI-*Z* (OR = 5.5, *p* = 0.01). Males with acceptable BMI-*Z* ( ≥0) were about four times more likely to have an FEV_1_%-predicted >80% (OR = 3.99, *p* = 0.02) after adjustment for age.

Body mass index-*Z* was also positively associated with age-adjusted FVC%-predicted in males (partial β-coefficient = 4.6, *p* = 0.02) after adjustment for age. Age-adjusted LBMI-*Z* was positively associated with FVC%-predicted (partial β-coefficient = 6.8, *p* < 0.0001), but again FMI-*Z* was not associated with FVC%-predicted (*p* = 0.16).

## Discussion

Body mass index is typically used in the clinical care setting of CF to assess nutritional status. In this study of generally healthy youth with PI-CF, LBMI was more strongly associated with pulmonary function than BMI was (particularly in males), while FMI was not associated with pulmonary function. These findings support the hypothesis that the effect of better nutritional status in CF is mediated through muscle mass, not FM, and that the association of total body mass with pulmonary function in CF reflects an effect of LBM. However, CF is also associated with altered body composition: individuals with CF have lower LBM than otherwise healthy individuals with similar BMI-*Z*.

Lower LBM may result in respiratory muscle impairment of the diaphragm or accessory respiratory muscles and lead to poorer lung function (8, 23–25). Especially concerning would be the loss of muscles involved in coughing, e.g., abdominal musculature. Alternatively, chronic pulmonary inflammation and infection result in a catabolic state, and lower LBM may reflect worse lung disease (10). Systemic inflammation and malabsorption in CF lend to protein and muscle loss (26). Decreased muscle is compounded by decreased physical activity (27), inflammation (28), and chronic glucocorticoid therapy (29).

Visceral fat is thought to contribute to metabolic abnormalities by secreting inflammatory adipokines (30–33). A predisposition toward accumulating FM in preference to LBM may induce additional inflammation in CF patients. This study did not distinguish between visceral and subcutaneous fat, and, thus, this relationship of visceral fat to pulmonary function cannot be directly tested here.

Among individuals with moderate to severe CF lung disease, pancreatic insufficiency, and nutritional failure, improvements in nutritional status are associated with improved lung function (2, 34). The benefit of targeting BMI ≥ 50‰ is clear, and predictive data from the logistic regression models in this study show a high likelihood of having normal lung function when BMI-*Z* ≥ 0. Better expectations can be made for patients with LBMI-*Z* ≥ 0.

In otherwise healthy individuals, increases in BMI at lower BMI generally reflect increases in LBM (35). This expected positive relationship between LBM and BMI seen in healthy individuals may be disrupted by inflammation in chronic illnesses (35). In our study, subjects with CF had lower LBMI-*Z* compared to controls with similar BMI-*Z*. Moreover, subjects with CF had greater FMI-*Z* compared to controls with similar Weight-*Z*, but this accumulation of fat over LBM was not reflected in our BMI-*Z* models. Instead, the relationship between FM and BMI-*Z* was similar to that observed in controls: curvilinear such that at BMI-*Z* > 1, there is proportionately greater FM (36), a relationship that was not altered by the presence of CF.

Sex differences are also important. In females with BMI < 50th‰, lower LBMI was associated with worse pulmonary function, whereas lower LBMI was associated with worse pulmonary function in males regardless of whether BMI was greater than or less than 50th‰. For two males with the same BMI, the male with lower LBM will have lower pulmonary function regardless of whether or not the BMI is in the recommended ≥50th‰ range (Table 2). In females, then, focusing on intervening in patients with BMI < 50th‰ appears particularly relevant (Table 2). In contrast, focusing on males with BMI < 50% may miss an important target group in the acceptable BMI range.

Accrual of lean muscle mass is the logical aim of nutritional and physical therapies. Aerobic fitness and survival in CF correlate closely (37) and exercise training and habitual physical activity improve muscle strength (38, 39), quality of life (40), and mucus clearance (41). The extent to which physical activity impacted body composition and pulmonary function in the subjects in this study was not explored.

Changes in body composition occur in males and females during puberty (42). Males predominantly gain FFM while females gain FM. In males, less LBM might reflect less physical activity, worse disease, or lower androgen effect, all of which may result from CF disease (43). However, our findings persisted even after adjustment of our models for puberty in both males and females.

The extent to which the results of this cross-sectional study of a relatively healthy CF population (mean FEV_1_%-predicted 89 ± 21) can be generalized to CF patients with poor lung function and co-morbidities including severe liver disease and CF-related diabetes is not clear. Additionally, longitudinal data are needed to assess the temporal relationship between LBM and lung function and to examine LBM and FM accrual in CF. Until recently, indices for FM and LBM have been unavailable outside the research setting. Wells et al. recently published new body composition reference data for DXA and the four-compartment model (44), and Weber et al. published LMI and FMI reference data based upon a sample from NHANES (45). The availability of this reference data may permit extension of these measures to the clinical setting.

Dual x-ray absorptiometry scanning is becoming increasingly appealing due to widespread availability, its accuracy in measurements (46, 47), and non-invasive nature. The 2005 Cystic Fibrosis Foundation Consensus Conference (48) recommends screening for decreased bone mineral density using DXA on select patients. While these recommendations are targeted to assess bone structure and health, in certain individuals, especially underweight females and males with low lung function suspected to be due to poor nutrition, assessing LBM via DXA may identify LBM depletion and could guide appropriate interventions. Recommendations for obtaining screening DXA scans to assess body composition will require more definitive longitudinal data that support its utility over anthropometry alone. Furthermore, large studies examining the effects of interventions on body composition are needed to pave the way for guidelines regarding the CF population at risk for fat and LBM deficits. At this time, BMI remains a good reflection of nutritional status although it may not fully depict the altered body composition we identified in CF.

In conclusion, we confirm that LBMI-*Z* is associated with FEV_1_%-predicted and that LBM is lower than one would expect for a given BMI. Thus, while BMI remains an adequate screening tool in CF patients, determination of LBM may be useful for assessing the relationship of body composition to pulmonary function particularly at lower BMI and in the clinical research setting; future interventions in CF may better examine the impact upon LBM with the use of DXA.

## Conflict of Interest Statement

The authors declare that the research was conducted in the absence of any commercial or financial relationships that could be construed as a potential conflict of interest.
